# Evidence-based dental management strategies for individuals with congenital hemophilia: a systematic review

**DOI:** 10.1186/s12903-026-07736-6

**Published:** 2026-01-30

**Authors:** Mathangi Kumar, Sulochana Badagabettu, Keerthilatha M Pai, Linu Sara George

**Affiliations:** 1 Department of Oral Medicine & Radiology, Manipal College of Dental Sciences, Manipal Academy of Higher Education, Manipal, Karnataka 576104 India; 2 Department of Fundamentals of Nursing, Manipal College of Nursing, Manipal Academy of Higher Education, Manipal, Karnataka 576104 India; 3 Department of Dental Surgery, Sikkim Manipal Institute of Medical Sciences, Sikkim Manipal University, 5th Mile Tadong, Gangtok, Sikkim India

**Keywords:** Hemophilia, Classic hemophilia, Dental prophylaxis, Hospital dental service, Access to healthcare

## Abstract

**Background:**

People with hemophilia commonly present with a range of bleeding symptoms, from minor oral cavity bleeds to life-threatening events such as intracranial hemorrhage. People with hemophilia often exhibit poor oral health, attributed to disease-specific barriers and a fear of dental procedures, which may exacerbate their oral disease burden. This systematic review aimed to evaluate the various dental procedures performed in patients with hemophilia and the corresponding clotting factor concentrate replacement regimens used for individuals with mild, moderate, and severe forms of the disease.

**Methods:**

An electronic database search was carried out using MEDLINE by PubMed, Scopus, Google Scholar, Web of Science, ProQuest, CINAHL, EBSCO Dental collection and EMBASE databases from inception to March 2025 for studies published in English language. The search strategy terms included were “hemophilia A”, “hemophilia A”, “hemophilia B”, “hemophilia B”, “dental care”, “oral surgery”, “complications”, “bleeding”. Case control studies, randomized controlled trials, observational studies and case reports addressing the dental treatments for people with hemophilia A/hemophilia B were included. Cases of acquired hemophilia and cases of hemophilia who are inhibitor positive were excluded.

**Results:**

A total of 23 studies were included for the final review. Majority of the studies have ensured to achieve 30% to 50% factor correction for dental procedures. Routine dental care procedures like restoration and dental scaling with the necessary technical specifications can be safely performed. Dental extractions can be safely performed using local infiltration anesthesia; however, administering nerve blocks typically necessitates prior administration of clotting factors due to the elevated bleeding risk. Adjuvant systemic anti-fibrinolytics like Epsilon-aminocaproic acid (EACA) and tranexamic acid greatly reduces the need for additional factor infusion.

**Conclusions:**

The use of prophylactic clotting factors during dental interventions in hemophilia patients showed notable inconsistencies across studies. This inconsistency underscores the importance of conducting larger, prospective clinical studies to better understand the rationale behind clotting factor use and its effects on dental treatment outcomes in patients with varying degrees of hemophilia severity.

**Supplementary Information:**

The online version contains supplementary material available at 10.1186/s12903-026-07736-6.

## Background

Hemophilia is the most common inherited bleeding disorder, caused by reduced levels of factor VIII (hemophilia A/classical hemophilia) or factor IX (hemophilia B/Christmas disease). A decrease in circulating factor levels leads to variability in disease severity (mild, moderate, or severe) and in the frequency of bleeding episodes. These episodes are typically managed by the exogenous administration of clotting factors concentrate under the supervision of healthcare providers [1]. The chronic and lifelong nature of the disease results in various complications and challenges in the daily lives of patients, thereby necessitating comprehensive care [2, 3].

People with hemophilia (PwH) commonly present with a range of bleeding symptoms, from minor oral cavity bleeds to life-threatening events such as intracranial hemorrhage [4]. Management of such episodes often requires hospital admission and monitored care, including the administration of plasma-derived substitutes to control bleeding. In the early 1950s, blood products such as fresh frozen plasma and cryoprecipitates were the mainstay for managing bleeding episodes [5–8]. However, advances in hemophilia care have led to a broader range of systemic treatment options, including plasma-derived and recombinant clotting factor concentrates, as well as non-factor therapies such as the bispecific monoclonal antibody Emicizumab [9].

A recent study demonstrated that gene therapy for hemophilia A, using lentiviral vector-transduced autologous hematopoietic stem cells, resulted in stable expression of factor VIII activity [10]. Clotting factor concentrates are currently classified as standard half-life (SHL), extended half-life (EHL), and ultra-long half-life (UHL) products [1]. The duration of these therapies in circulation allows for safer and more effective management of complications. Treatment strategies vary between prophylactic and on-demand factor therapy, contributing to differences in disease manifestation and severity [11].

People with hemophilia often exhibit poor oral health, attributed to disease-specific barriers and a fear of dental procedures, which may exacerbate their oral disease burden [12]. Routine dental treatments pose a significant bleeding risk in these individuals, with potential for delayed and prolonged bleeding that can be difficult to manage for both clinicians and patients. Dental treatment protocols for PwH vary globally, and there are currently no universally accepted guidelines applicable in clinical settings [13]. This variability has led to reluctance among dental care professionals to perform procedures on PwH [14, 15].

Most dental studies involving PwH are retrospective and based on medical records; prospective studies evaluating dental treatment outcomes, particularly stratified by disease severity, are scarce [13, 16–19]. The heterogeneity in dental management protocols highlights the need to analyze existing clinical evidence from a dental care perspective.

Therefore, this systematic review aims to evaluate and analyze the amount of clotting factor replacement used for various dental treatments in individuals with mild, moderate, and severe hemophilia.

## Methods

### Research question

This systematic review was registered in PROSPERO CRD42025635460. This review was conducted following the Preferred Reporting Items for Systematic Review and Meta-Analyses (PRISMA) guidelines and according to the Participants, Interventions, Control, Outcomes, Study design (PICOS) principles. The addressed research question was: “What are the various dental treatments performed in patients with hemophilia and what is the amount of clotting factor concentrate replacement therapy prescribed for various dental procedures in people with mild, moderate and severe hemophilia?”

### Eligibility criteria

#### Population

Studies addressing the various dental treatment procedures that were performed for people with congenital mild, moderate or severe hemophilia A/ hemophilia B (exclusive hemophilia cohort) were included. Studies addressing other bleeding disorders (von Willebrand disease, platelet function disorders, other coagulation disorders) along with hemophilia were excluded. Acquired hemophilia and inhibitor positive hemophilia were excluded.

#### Intervention

The amount of clotting factor replacement therapy employed pre, during and post dental treatment procedures for people with mild, moderate and severe hemophilia. The various dental procedural modifications employed pre, during and post dental treatment procedures for people with mild, moderate and severe hemophilia.

#### Comparator(s) or control(s)

No specific comparator groups.

#### Outcome

The reported prophylactic factor concentrates used for dental treatment procedures, anti-fibrinolytic drugs employed, and the dosages of clotting factor utilized for dental procedures, post-procedural measures to control bleeding, special technical modifications adopted, outcomes in terms of complications of the dental treatment procedures.

#### Study design

All observational studies, case-control studies, cohort studies, randomized controlled trials, case reports addressing the various dental treatment procedures that were performed for people with congenital hemophilia A/ hemophilia B were included.

### Study selection

A literature search of PubMed/MEDLINE, Embase, Scopus, and Web of Science, CINAHL, EBSCO Dental Collection, ProQuest was conducted to identify all relevant studies published up to 31st March 2025 in English language, using the following keywords: hemophilia A/B, hemophilia A/B, oral surgery, dental treatments with Boolean operators (AND, OR). The search strategy terms included were “hemophilia A”, “hemophilia A”, “hemophilia B”, “hemophilia B”, “dental care”, “oral surgery”, “complications”, “bleeding”.

One reviewer (Author MK) evaluated the included reports and excluded the unrelated reports and duplicate articles. Of the 873 articles that were identified, 58 articles were screened and the grey literature on this topic was searched from relevant sources to ensure comprehensive search. Duplicate detection of articles was performed through Rayyan^®^ software. Two reviewers (MK, SB) systematically evaluated the article for the title, abstract as well as the full text and entered the details in the Microsoft Excel data sheet. Any disagreements between reviewers during study selection or data extraction were resolved after consultation with a third reviewer (Author: LSG). The articles included were then screened for the full text and the data were summarized into tabular format.

### Quality assessment

The Joanna Briggs Institute (JBI) critical appraisal checklist for observational and cohort was used for quality assessment of the included studies [1]. Two reviewers (MK, SB) performed this task. Studies were restricted to only the dental management of PwH. Quality assessment was performed to note the details regarding the pre-dental treatment factor coverage and relevant systemic anti-fibrinolytic regimen that was employed along with the takeaway lessons from the included studies.

### Data extraction

For each study the following data were extracted: authors, year and country of the study, study design, amount of clotting factor used (prior, during and after dental procedure), type of dental procedure, pharmacological considerations (type of anesthesia, analgesics etc.) description of the participants (sample size, mean age), follow-up findings, systemic antifibrinolytic therapy, local hemostatic agents used clinical complications and outcomes.

## Results

### Study selection

**Fig. 1:**
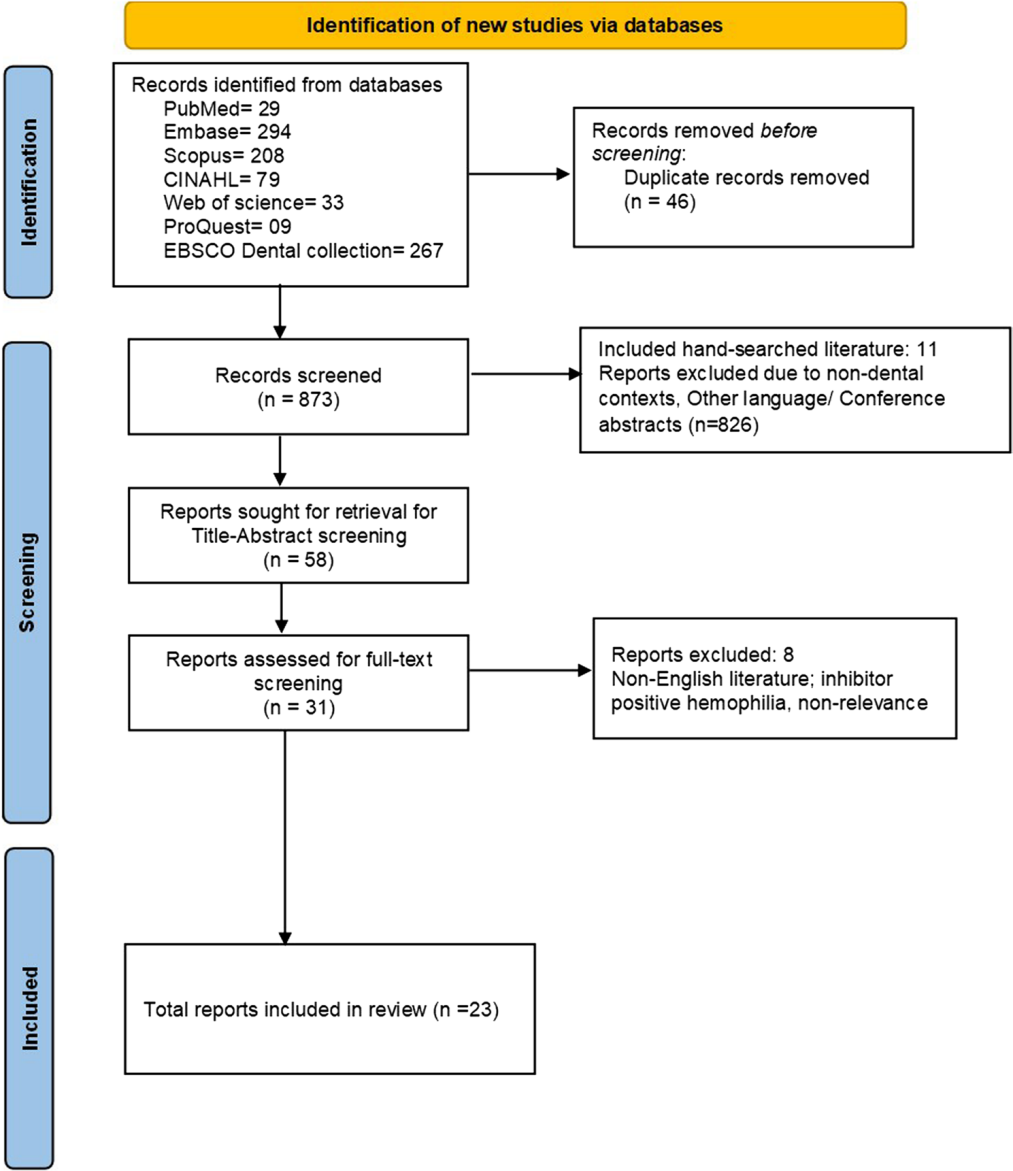
PRISMA flowchart depicting the selection of included studies

Online search yielded a total of 873 studies (including eleven additional studies through hand-search) (Fig. 1). After duplicates removal (46 articles), 58 publications remained, of which 27 studies were found to be irrelevant and were thus excluded. A final full-text review was conducted on the remaining 23 studies.

## General characteristics of the included studies

Three randomized clinical trials (RCT) comprising were included [2–4]. Studies were conducted in United Kingdom [2, 5–7], United States of America [8–10], Europe [11–17], Asia [3, 18–21] and Australia [22, 23] and Africa [4, 24]. The characteristics of the included studies are depicted in Table 1. All studies included only PwH with mild, moderate or severe variants. The amount of clotting factors concentrates administered prior and post dental treatment was synthesized from the included studies. Dental treatment procedures like dental scaling, restoration of teeth, surgical extractions and the corresponding amount of factors prior to and post-procedure from each included study was documented and is depicted in Tables 2 and 3.

**Table 1:** Table depicting the study characteristics and outcomes

S.No	Author, year, study design	Sample size and type of Hemophilia [A/B]	Procedure type	Anesthesia used	Follow-up details	Reported complications
1.	Middleton D S et al. (1965, United Kingdom) [5]	Hemophilia A: 28 (mild: 8; moderate: 5 and severe:15); Hemophilia B: 8 (mild:7, severe:1)	36 dental extractions in pediatric and adults (7 to 44 years)	Local infiltration anesthesia for dental extractions; No block anesthesia	In some cases, local hemostatic action proved inadequate to arrest this hemorrhage and further transfusion was administered.	Delayed post-op bleeding (Day 7 and Day 9) which is within 48 h following the cessation of transfusion.
2.	Needleman et al. (1976, USA) [8]	11 subjects with Hemophilia A; 7 to 25 years	Sixty-six dental procedures—13 odontectomies (surgical removal of impacted teeth), 18 forceps extractions	Nitrous oxide-oxygen and halothane general anesthesia; lidocaine hydrochloride (Xylocaine 2%) with epinephrine 1/100,000 local anesthesia.	Not mentioned	Most serious bleeding occurred within the first 24 h postoperatively in one patient which was well controlled with pressure and the placement of additional sutures
3.	Walsh et al. (1971, United Kingdom) [6]	Unclear data	Dental extractions	Not mentioned	Not mentioned	Not mentioned
4.	Forbes C D et al. (1972, Kenya) [4]	Randomized controlled trail Twenty-eight patients aged 13 to 65 years were studied. 20: Hemophilia A and 8 patients with Hemophilia B.	32 separate episodes of dental extraction.	Not mentioned	Not mentioned	Not mentioned
5.	Tavenner R W H et al. (1972, UK) [7]	Sample size: 19 patients with hemophilia A and 3 with hemophilia B	Dental extractions	Not mentioned	Not mentioned	Not mentioned
6.	Bjorlin G et al. (1973, Europe)[11]	7 with hemophilia A and five with hemophilia B	Dental extractions	Not mentioned	Not mentioned	Not mentioned
7.	Stephen E et al. (1983, USA) [10]	16 patients 11 patients had hemophilia A and five had hemophilia B	Dental extractions Sixteen patients underwent 19 surgical procedures for the extraction of 35 permanent (32 molars, 3 incisors) and 10 deciduous teeth.	Not mentioned	Not mentioned	Not mentioned
8.	Zanon et al. (2000, Europe)[13]	75 adults with hemophilia A/B (32 ± 14 years).	45 of 98 (45.9%) dental extractions under local anesthesia in hemophilia patients were surgical extractions	Not mentioned	Not mentioned	Outcome: Delayed bleeding 2 days after extraction of the third inferior molar.
9.	Xavier Frachon e t al. (2005, Europe)[16]	55 dental extraction	Hemophilia A: Severe: 6 cases (6 interventions)—13 extractions Moderate: 1 case (3 interventions)—14 extractions; Mild : 4 cases (4 interventions)—8 extractions; Hemophilia B: Moderate: 1 case (3 interventions)—13 extractions; Mild hemophilia B: 2 cases (2 interventions)—3 extractions	Not mentioned	Not mentioned	Not mentioned
10.	I. Hewson et al. (2011, Australia) [25]	Hemophilia A: 26 subjects; Hemophilia B: 11 subjects	Hemophilia A: Severe: 9 (three were on regular prophylaxis; 13–19 IU kg three times a week, six were on demand factor support); Moderate: 5(1–5% FVIII activity); Mild in 12 patients (> 5% FVIII activity); Hemophilia B: Severe, moderate: 1 each; Mild: 9	Dental extraction procedures under general or local anaesthesia with 27-gauge single use needle, aspiration was used to ensure the needle was not in a vessel 1:200 000 adrenaline via infiltration and/or inferior alveolar nerve block as required	Not mentioned	Not mentioned
11.	Hewson ID et al. (2010, Australia)[22]	32 year old male with severe hemophilia B	Maxillary and mandibular third molars surgically removed under general anesthesia	General anesthesia	Not mentioned	Not mentioned
12.	Rayen R et al. (2011, India)[18]	4-year-old child with mild hemophilia	multiple dental treatments: Full mouth rehabilitation	General anesthesia	Not mentioned	Not mentioned
13.	Andre Peisker et al. (2014, Europe)[17]	58 patients with Hemophilia A and B	Dental extraction; 15 patients during 19 interventions	Not mentioned	Not mentioned	Two patients presented postoperative bleeding. One had secondary bleeding requiring additional factor concentrates.
14.	Givol N et al. (2015, Israel) [21]	Hemophilia A: 84 (Mild:16; Moderate:6; Severe:70); Hemophilia B: 25 (Mild:1; Moderate:9; Severe:15)	125 patients undergoing a total of 1968 dental procedures,	Local anaesthesia (lidocaine 2% with adrenaline 1:100 000) inferior alveolar nerve block under factor cover or local infiltration at the operative site	Not mentioned	Bleeding was defined as excessive bleeding during or within 20 days following the dental procedure and 40 bleeding events were documented of 1968 procedures in 125 patients
15.	SP Mahlangu1 et al. (2018, South Africa) [24]	Hemophilia A: 10 subjects ; Hemophilia B: 3 subjects ; Age range: 3 to 61 years	Dental extractions accounted for 58% of dental procedures performed	Not mentioned	Not mentioned	Post-extraction bleeding complications in two patients with severe hemophilia managed with intra-venous factor replacement and tranexamic acid orally and mouthwash.
16.	Simona Raso et al. (2022, Europe) [15]	107 patients with only mild hemophilia (Age range: 18–81 years)	38 patients underwent 42 different dental procedures: 22 single and three multiple dental extractions, seven teeth cleanings, seven dental caries, and three oral surgeries	Not mentioned	Not mentioned	Not mentioned
17.	Emma Fribourg et al. (2024, France)[14]	Not mentioned	Oral surgical procedures	Not mentioned	Not mentioned	Not mentioned
18.	N Pai et al. (2024, India) [19]	54 patients; 42 hemophilia A and 10 hemophilia B	Tooth extraction and soft tissue management as minor surgeries, while all procedures performed under general anesthesia were considered major surgeries.	Local and general anesthesia	Not mentioned	Not mentioned

**Table 2:** Table depicting the clotting factor replacement therapy/ hemostatic cover employed for the dental treatment procedures

S.No	Author, year, study design	Pre and post procedural Prophylactic clotting factor concentrate for dental procedures / Hemostatic measures used for dental procedures	Other details
1.	Middleton D S et al. (1965, United Kingdom) [5]	Preop regimen: Plasma derived factor or fresh frozen plasma as substitutes to raise Factor VIII level (10 to 60% increase) Post-op regimen: up to 7 days postoperatively by giving, every 6 hourly plasma transfusions.	The dried socket was then packed gently with thrombin, the protective splint was fitted, and the accuracy of fit was carefully checked Atraumatic silk suture was inserted to approximate the mucosa where buccal bone had been removed Splint was removed after 48 h (earlier if necessary) and checked to make sure that no pressure points had developed.
2.	Needleman et al. (1976, USA) [8]	Pre-op regimen: One hour before treatment, the patient received 40 units/kg of factor V III as cryoprecipitate or as a commercial concentrate of factor V III. This dose raised the patients’ factor VIII level to approximately 80% of normal. Epsilon-aminocaproic acid, 100 mg/kg, in normal saline solution was then administered intravenously. Post-op regimen: Factor VIII (units) intravenously, 40 units/kg day 5 and 20 units/kg bid for 10 days	Not applicable
3.	Walsh et al. (1971, United Kingdom) [6]	Plasma derived factor concentrates Preoperative factor levels corrected to 50% Mean dosage of factor: 231.5 IU/kg/patient Mean number of doses: 11.5 All patients received **6** g of Epsilon-aminocaproic acid (EACA) by intravenous infusion within ***2*** hr prior to dental extraction followed by orally administered EACA **6** g four times daily, beginning **6** hr after completion of the surgical procedure and continuing for an average of approximately **10** days	Not applicable
4.	Forbes C D et al. (1972, Kenya) [4]	Double-blind technique and random allocation to the trial the patients received either tranexamic acid (1 g three times a day) or placebo tablets that was administered two hours before extraction and continued for five days. Tranexamic acid, 1 g three times a day for five days, significantly reduced blood loss and transfusion requirements after dental extraction in patients with hemophilia A and B	Not applicable
5.	Tavenner R W H et al. (1972, UK) [7]	Blood and plasma as substitutes which were used for the control of bleeding after dental extraction along with tranexamic acid. The results with tranexamic acid was better than those obtained with aminocaproic acid; the dose used was lesser with fewer and side effects with tranexamic acid.	Not applicable
6.	Bjorlin G et al. (1973, Europe) [11]	Administration of a single dose of factor VIII or factor IX concentrate in combination with tranexamic acid (Cyclocapron 25 mg. per kilogram of body weight every 6 h) in association with tooth extractions may be recommended in patients with hemophilia A and B.	Not applicable
7.	Stephen E et al. (1983, USA) [10]	Preop prophylactic dose: Average initial dose: 11.9 U/kg 20–24% increase in the factor VIII level and a 10–12% increase in the factor IX level Postoperative dose: Average dose: 33.8 U/kg	Not applicable
8.	Zanon et al. (2000, Europe) [13]	Pre-op regimen: 20 mg/kg of tranexamic acid and a single infusion of factor VIII or IX to achieve a peak level about 30% of factor VIII or IX in vivo prior to dental extraction. Together with the replacement therapy the patients received 20 mg of tranexamic acid in 100 ml of saline solution i.v. Eight and 16 h later and in the following 7 days three times a day, 20 mg of tranexamic acid was administered p.o.	After extraction, pieces of fibrin sponge were inserted in the wound and a silk suture was applied in all hemophilia patients. Resorbable sutures were not used for two reasons: (i) the time of reabsorbtion of resorbable suture varies from type to type and depends on non-standardized factors, and (ii) the higher cost. A gauze saturated with tranexamic acid was maintained in place for 30 ± 60 min and an ice bag was placed on the cheek for the same length of time. No patients reported adverse events during antifibrinolytic treatment
9.	Xavier Frachon et al. (2005, Europe) [16]	50 IU/kg of recombinant factor was initiated in 6 out of the 7 patients with severe hemophilia A and 1 patient with moderate hemophilia A (planned for numerous extractions) 70 IU/Kg of recombinant factor IX for hemophilia B patients (3 cases) Local hemostasis, intermittent compression with oral tranexamic was prescribed for 3 days at the rate of one 10-minute compression using a saturated compress, repeated hourly on the first day, every 2 h on the second day, and every 3 h on the last day.	This protocol produced a reliable outcome, limiting the duration of the hospital stay to 24 h in most cases, and improving postsurgical comfort thanks to a combination of systemic treatment and local hemostasic measures including intermittent tranexamic acid compression.
10.	I. Hewson et al. (2011, Australia) [25]	Average factor utilized for delayed post-op bleeding: 2000 IU 5% tranexamic acid to not disrupt any clot formed, surgical was then placed in the socket containing the clot and tranexamic acid and the socket was tightly closed with 4 − 0 monocryl or 4 − 0 Vicryl	Not applicable
11.	Hewson ID et al. (2010, Australia) [22]	Postoperative complication at day 10: Oozing from both mandibular sockets managed with 5% tranexamic acid mouthwash, oral tranexamic acid (1 g three times a day). Local measures with 5% tranexamic acid solution, surgicel and monocryl sutures after a single preoperative dose of Factor IX. This socket was flooded with 5% tranexamic acid and Surgicel placed in the socket.	Not applicable
12.	Rayen R et al. (2011, India) [18]	200 Units as a preoperative loading dose by slow infusion, half an hour prior to treatment and 200 units as a maintenance dose 12 h postoperative. Surgicel* (Oxidized Cellulose) was used; No complications reported on follow up	Not applicable
13.	Andre Peisker et al. (2014, Europe) [17]	Replacement therapy with recombinant and plasma-derived factor VIII and IX was applied systematically in combination with antifibrinolytic treatment and local haemostatic measures Excellent haemostasis is achievable following a protocol using defined pre- and postoperative doses of factor concentrates in combination with haemostatic measures	Not applicable
14.	Givol N et al. (2015, Israel) [21]	Pre-op regimen: Antifibrinolytics (tranexamic acid, epsilon amino caproic acid) with injection of subcutaneous DDAVP (desmopressin; Factor replacement therapies – either factor VIII or IX concentrates (initial dose of 30–50 IU/kg) 1–2 h before performing dental procedures Local haemostatic measures included the use of silk sutures, gelatin sponges, gauze saturated with tranexamic acid and fibrin glue.	Factor transfusions are not mandatory and should be applied considering the procedure-related risk and the patient’s calculated haematological risk for bleeding
15.	SP Mahlangu1 et al. (2018, South Africa) [24]	Local haemostatic agents were used in 42% of the study population.	Not applicable
16.	Simona Raso et al. (2022, Europe) [15]	Haemostatic agents: Antifibrinolytic therapy oral tranexamic acid (1 g three-times-daily) alone and perioperative factor replacement to the absence of treatment at all Pre-op regimen: Almost all patients received a preoperative single dose of coagulation factor; Post-op regimen: Treatment of abscess incisions the treatment regimen involved a postoperative dose	Majority of oral interventions (27/42, 64%) were managed with clotting factor concentrates; four patients did not receive any hemostatic therapy for dental procedures.
17.	Emma Fribourg et al. (2024, France) [14]	Pre and perioperative use: Desmopressin (IV: MinirinR or nasal spray) FVIII concentrate slow preoperative IV injection of 30–50 U/kg at H-1. postoperatively, every 12 h from 1 to 4 times, at a dosage of 30 U/kg. Factor IX concentrate, either of x^®^ plasmatic origin (e.g., Betafact^®^) or recombinant (e.g. Benefix^®^) slow preoperative IV injection of 50–70U/kg at H-1. Repeat injections postoperatively, every 12 h from 1 to 4 times, with a dosage of 30–50 U/kg.	Absorbable collagen sponges and sutures were routinely placed in the surgical wound Surgical fibrin glue (TisseelR 2 mL) Tranexamic acid was prescribed Patients were given standard postoperative advice (dental hygiene and food instructions), and three times a day for 10 days.
18.	N Pai et al. (2024, India) [19]	Fibrin glue (Tisseel Lyo) has been used as a hemostatic method in patients undergoing invasive dental procedures.	Not applicable

**Table 3:** Table depicting the dental procedural modifications and hemostatic agents employed for moderately invasively dental treatment procedures (dental restorations, dental scaling procedures)

S.No	Author, Year, Country	Findings from the study
1.	Michaelides L Paul et al. (1983, USA) [9]	• Periodontal surgical procedures can be taken up on an outpatient basis • Patient preparation: To ensure 50% rise in plasma clotting factor levels one hour prior to the dental procedure • To stabilize the clot, periodontal packs are recommended. • Maintenance appointments to include oral hygiene review, scaling, and topical fluoride application without prior plasma clotting factor infusion
2.	Pedersen Sindet et al. (1988, Europe) [12]	• Sample size: 15 patients • Hemophilia A: 7 subjects; Hemophilia B : 8 subjects • Hemophilia severity data: Unclear • Comparative study on two interventions for gingival bleeding: Group I: EACA (Epsikapron’’)* administered in 4 daily oral doses of 100 to 200 mg/kg body weight during 4 d (2 sessions), or tranexamic acid (Cyklokapron^®^)*. administered orally 4 times daily in doses of 25 mg/kg for a minimum of 4 d (7 sessions). Group II: no replacement therapy but systemic and/or local antifibrinolytic treatment with tranexamic acid • Study concluded that treatment of gingival bleeding in hemophilic patients should include local antifibrinolytic treatment.
3.	Lee APH et al. (2005, UK) [2]	• Double-blind cross-over randomized control trial • Sample size: 13 patients • 11 subjects with hemophilia A and 2 subjects with hemophilia B. • Severity: one mild hemophilia, 3 moderate and 7 severe hemophilia A. 2 severe hemophilia B. • Experimental treatment regime (ETR): Intravenous placebo (0.9% saline) before scaling both quadrants on one side of the mouth followed by oral rinsing with Tranexamic acid mouthwash (TAMW) four times daily for up to eight days. • Control regime (CR): Each patient received Factor Replacement Therapy before scaling the opposite side of the mouth followed by use of a placebo TAMW. Each patient underwent both treatments in a randomized sequence. • No statistically significant difference was found in gingival bleeding and mouth washing frequencies between the groups
4.	Sivakumar Nuvvula (2014, India) [3]	• Randomized clinical trial; Mean age: 23.63 years (13.2 to 53.6 years) • Sample size: 19 patients: 12 were with hemophilia A (3 severe and 9 moderate) and 7 hemophilia B (3 severe, 3 moderate and 1 mild). • Experimental treatment regime (ETR) involved saline transfusion followed by freshly prepared tranexamic acid mouth wash (FTAMW) and the control treatment regime (CTR) involved factor replacement therapy (FRT) followed by placebo mouthwash. • The difference between the reported cases of bleeding in CTR and ETR was not significant statistically (*P* = 0.63).
5.	Chandra Khyati et al. (2016, India) [20]	• Periodontal therapy: Scaling and root planing: 30-year-old male with severe hemophilia A • Protocol employed for dental procedure: Tranexamic acid 10 • mg/kg in 20 ml normal saline infused preoperatively over 20 min (with 1 g every 8 h orally for 5 days postoperatively) with intravenous factor VIII replacement to build up 50–75% along with local hemostatic measures and tranexamic acid mouth rinse and factor VIII maintenance for 7 days and postoperative care. The patient weighed 70 kg and so 2800 IU/dl was infused one hour before the procedure to raise his factor VIII level by 80%. After 10 h, 1400 IU/dl was infused for next 3 days and tapered to 700 IU/dl for another 3 days. • Post treatment: Tranexamic acid mouthwash with 10 ml of 4.8-5% solution 4–5 times a day for 2 min for 2–5 days and was extended to 8 days.

The results of the JBI critical appraisal checklist for case reports and case series showed that the quality of reports included for this review were low to moderate. All the included reports scored 4 or higher out of the ten criteria. The reliability of the overall evidence was constrained by various methodological weaknesses identified within the included studies. It should be noted that studies performed on the dental aspects of hemophilia are relatively rare. In particular, a majority of the studies did not implement adequate measures to address the various factors into consideration for calculating the amount of prophylactic dose required for dental procedures. Also, the non-uniform method of reporting on follow-up duration was noted. These methodological issues introduce risks of bias and may compromise both the accuracy and generalizability of the results. To enhance the quality of future research, more rigorously designed cohort studies that follow established methodological and reporting standards are recommended.

The reported special treatment modifications adopted by various authors for invasive dental procedures are as follows:

Dental extraction and surgical removal of third molar teeth has been adequately evaluated in various studies with the anesthetic considerations to be employed. These aspects are depicted in Table 1. Routine dental care procedures like restoration and dental scaling with the necessary technical specifications were evaluated and depicted in Table 2. The local hemostatic agents used for the management of bleeding along with the systemic anti-fibrinolytic medications has been depicted in Tables 1 and 2.

Majority of the studies have ensured to achieve 30% to 50% factor correction for dental procedures as per the World Federation of Hemophilia guidelines [6, 13, 19, 25]. The dose of factor that is to be given for each case is calculated using the following standardized formula as follows [26, 27]:

*Dose (IU) of Factor VIII = body surface (kg) x level of desired factor VIII increase (IU/dL) x 0.5*.

*Dose (IU) of Factor IX = body surface (kg) x level of desired factor IX increase (IU/dL)*.

## Discussion

Comprehensive care model is an effective mode for hemophilia care. It involves a multidisciplinary approach, typically provided through hemophilia treatment centers or comprehensive care facilities. These centers bring together a coordinated team—often including a hematologist, pediatrician, nurse, dentist, physiotherapist, and psychosocial specialist—to address the diverse medical and psychosocial needs of individuals with hemophilia. Such collaborative care has been shown to enhance overall health outcomes and significantly reduce disease-related complications [28, 29].

A strong collaboration between hematologists and dental professionals within this care framework can increase patient confidence in accessing routine dental services. Integrating regular dental assessments and timely interventions as a core element of comprehensive care is essential. This patient-centered model should ideally be implemented from the level of primary prevention to support long-term oral and systemic health in people with hemophilia [30].

The management of dental patients with hemophilia necessitates meticulous interdisciplinary collaboration to ensure procedural safety and optimal clinical outcomes. Prior to any invasive intervention, comprehensive consultation with the patient’s hematologist is imperative to evaluate bleeding risk, determine the requirement for factor replacement therapy, and establish appropriate treatment scheduling. A coordinated, multidisciplinary approach involving dental surgeons, hematologists, and anesthesiologists facilitates the maintenance of adequate hemostatic control while minimizing procedural complications. Detailed documentation of the patient’s bleeding history, inhibitor status, and prophylactic regimen is essential to guide individualized treatment planning. In collaboration with hematologists, dental professionals should also consider the adjunctive use of antifibrinolytic agents, such as tranexamic acid, to enhance local hemostasis and reduce postoperative bleeding. Postoperative management should involve clear interprofessional communication regarding pain control and follow-up, with the avoidance of analgesic medications that interfere with platelet function. Furthermore, the integration of preventive oral health strategies within the multidisciplinary care framework can reduce the need for invasive procedures and mitigate bleeding-related risks. Regular interdisciplinary case discussions and the establishment of standardized referral pathways strengthen coordination between dental and hematology services, thereby promoting evidence-based, patient-centered care for individuals with hemophilia [31, 32].

Dental management of people with hemophilia is quite complex and literature evidence on this aspect is barely studied. Systematic review on the analysis of case reports across the globe suggests that there is a vast diversity in the protocol employed for dental management for PwH [27]. Moreover, guidelines proposed by various professional organizations and clinical settings suggest offering dental care under supervised tertiary care centers [25, 33]. Data on the amount of factors to be administered suggested by these guidelines are non-uniform across the severity of hemophilia and the type of dental treatment that is performed on PwH [27]. Also, the risk of bleeding is dictated by the nature of the dental procedure which can result in varying degrees of hemorrhage [34]. Neto R et al. (2021, South America) [35] reported a case of mild hemophilia B who was diagnosed following bleeding complications after five days of extraction.

The variation in the prophylactic factor therapy employed for the dental procedures can be attributed to the mode of care wherein prophylaxis versus on demand factor administration [27]. Also, studies do not specify the type of clotting factor concentrate employed (standard half-life versus extended half-life products). Also, the clinical setting in which the treatment was planned (dental clinical setting versus comprehensive setup) and the exact details of the dental treatment type. Moreover, since the factor dose is determined by the body weight, pediatric and adult dosages cannot be compared. Studies have not clearly depicted these aspects. The comprehensive care of management approach with enhanced access to care for PwH significantly reduces the need for hospitalization and treatment burden [28].

Authors have evaluated the use of specialized splints for stabilizing the hematoma formed in tooth extraction sockets [5, 36]. The function of these splints is to prevent the movement and undue pressure on the socket or adjacent mucosa following dental extraction [5]. High-risk dental treatment procedures warrant the need for prophylactic factor infusion wherein some of the authors advocate the use of single pre-op dose mandatory for all patients irrespective of the severity of hemophilia [5, 11, 13, 15, 23]. However, the details of the outcomes from these studies have been barely addressed. An analysis of clinical practices by Kumar M et al. for managing dental procedures in individuals with hemophilia within a comprehensive tertiary care framework highlighted the critical need for standardized protocols on prophylactic administration of clotting factor to effectively minimize the risk of bleeding complications [32].

Local infiltration anesthesia with adrenaline in the area interest can be safely administered without reported complications. However, inferior alveolar nerve block anesthesia requires clotting factor infusion due to the deep intramuscular penetration of the needle, which carries a higher risk of bleeding [5, 8, 21, 22]. General anesthesia can be safely administered, and dental procedures can be performed without any additional risk in PwH [8, 18].

Local hemostatic agents like surgicel, gel-foam, thrombin powder has been utilized in the studies for achieving hemostasis following dental procedures [5, 15, 19]. The use of combination of factor therapy along with effective anti-fibrinolytic medications (epsilon amino caproic acid, tranexamic acid tablets) have been extensively studied by authors which have proven to be effective [4, 6, 13, 16, 25].

A retrospective cohort study from Japan with 151 extractions in 84 interventions among 55 patients with hemophilia, reported that the incidences of post-extraction bleeding per intervention and per tooth extraction were 11.9% and 7.9%,respectively, and that the use of mouth splints significantly reduced the risk [36]. Aruche et al. (2022, Europe) [37] reported dental extraction of right premolar in a patient with severe hemophilia A on emicizumab prophylaxis. Extraction under articaine 4% adrenaline local anaesthetic with only tranexamic acid and local measures was performed to manage post-operative bleeding. There was minimal bleeding post-operatively with the patient maintaining a stable blood clot and requiring no further intervention. Nadia Cocero et al. (2014, Europe) [38] suggested the use of autologous plasma rich in growth factors instead of fibrin glue in the prevention of severe bleeding after teeth extractions in patients with congenital bleeding disorders.

The plausible reason for delayed bleeding is because the levels of factor VIII tends to fall to the basic level within 24 h after cessation of transfusion and, unless healing is sufficiently advanced, bleeding may then recur. This warrants a close monitoring of the surgical site in the first few days following the procedure [39].

Complications associated with highly invasive dental procedures in patients with hemophilia can be substantially minimized through the implementation of a carefully designed hemostatic management plan. The plan should ensure adequate clotting factor replacement coverage during the perioperative period, including a sufficient post-procedural duration. Delayed postoperative bleeding in hemophilia typically occurs around the fifth day after the procedure, and results from impaired secondary hemostasis which is characteristic of the disorder. An effective strategy may include administration of a single factor replacement dose on the first postoperative day, combined with adjunctive oral tranexamic acid therapy for up to five days, to significantly reduce the risk of delayed bleeding and other hemorrhagic complications [32]. This regimen has proven to be clinically effective in the included studies in this systematic review [40–42].

Rationale and careful optimization of clotting factors concentrate during dental care can lower treatment expenses and reduce the need for additional interventions. Timely dental interventions help minimize the economic burden and prevent unnecessary hospitalizations. Therefore, emphasizing the importance of prophylactic dental care in patients with hemophilia is warranted. Employing atraumatic techniques, along with local hemostatic agents, contributes to a safer and more effective dental treatment experience.

The various guidelines for hemophilia management exhibit variability in their development methodologies based on the model of care offered by the country. The World Federation of Hemophilia (WFH) has attempted to harmonize these recommendations by accounting for the diversity in global healthcare systems. However, the heterogeneity in hemophilia management practices has resulted in differing expert perspectives tailored to specific clinical environments, making it difficult to evaluate their relevance to dental care in individuals with hemophilia. Furthermore, disparities in the availability of clotting factor concentrates (CFC) and access to healthcare services present additional obstacles to the practical implementation of these guidelines [27, 32].

The limitations of the present study include the heterogenous nature of treatment protocols employed owing to which the findings cannot be generalizable and meta-analysis was not possible. Further, the outcomes from these studies in terms of objective bleeding rates, adverse events and patient reported outcomes are not addressed in the included articles. The treatment regimen used was consistent with the World Federation of Hemophilia guidelines; however, the authors did not specify the type of extractions performed and the duration of factor administration in their study, making it unclear whether the procedures fell under the category of minor or major surgery [17]. The outcomes of such dental procedures that are performed by employing a standardized approach need documentation in literature since the complications that arise from dental procedures are multifold. Authors have interpreted observations and results from studies across all severities of hemophilia, regardless of differences in factor levels. Such extrapolations may result in ambiguity regarding the appropriate treatment regimens for each severity level [2, 3]. Studies have been performed in patients, included under a broad umbrella of inherited bleeding disorders and the dental perspective has been extrapolated in a vague manner with lack of reporting specific to individual disorder specifying hemophilia etc. Thus, the details regarding the dental treatment procedures are underreported resulting in lack of clarity [14]. The need for a collaborative and interdisciplinary approach to seek the hematologist consultation for the dose of prophylactic factor has not been addressed in the studies.

## Conclusion

The findings of this review demonstrate considerable variability in the dosage of clotting factor administered during various dental procedures in individuals with hemophilia. Therefore, there is a need for further evidence-based, prospective clinical studies focusing on the dental management of patients with congenital hemophilia, along with documented clinical outcomes, to establish standardized recommendations for clotting factor concentrate dosing to ensure the safety of dental interventions.

### Protocol and registration

The present review adhered to the guideline outlined in PRISMA and PRISMA-P checklist. The review protocol is registered at the International Prospective register of Systematic Reviews (PROSPERO) database under the protocol number CRD42025635460.

## Supplementary Information

Supplementary Material 1.

Supplementary Material 2.

## Data Availability

The dataset supporting the conclusions of this article is included within the article. However, additional information can be requested from the corresponding author upon reasonable inquiry.
